# Early Brain Injury After Poor-Grade Subarachnoid Hemorrhage

**DOI:** 10.1007/s11910-019-0990-3

**Published:** 2019-08-29

**Authors:** Verena Rass, Raimund Helbok

**Affiliations:** Department of Neurology, Medical University of Innsbruck, Anichstrasse 35, 6020 Innsbruck, Austria

**Keywords:** Subarachnoid hemorrhage, Early brain injury, Neuroimaging, Neuromonitoring, Treatment

## Abstract

**Purpose of Review:**

Over the last years, the focus of clinical and animal research in subarachnoid hemorrhage (SAH) shifted towards the early phase after the bleeding based on the association of the early injury pattern (first 72 h) with secondary complications and poor outcome. This phase is commonly referenced as early brain injury (EBI). In this clinical review, we intended to overview commonly used definitions of EBI, underlying mechanisms, and potential treatment implications.

**Recent Findings:**

We found a large heterogeneity in the definition used for EBI comprising clinical symptoms, neuroimaging parameters, and advanced neuromonitoring techniques. Although specific treatments are currently not available, therapeutic interventions are aimed at ameliorating EBI by improving the energy/supply mismatch in the early phase after SAH.

**Summary:**

Future research integrating brain-derived biomarkers is warranted to improve our pathophysiologic understanding of EBI in order to ameliorate early injury patterns and improve patients’ outcomes.

## Introduction to the Concept of EBI

Aneurysmal subarachnoid hemorrhage (SAH) accounts for 5–7% of all stroke types [1] and primarily affects young patients at their most productive years. Despite improved neurocritical care management with a decrease in case fatality over the last decades, SAH is still a devastating disease with high long-term morbidity [2]. Only two-thirds of survivors regain functional independence at 1 year after the bleeding [3]. Mechanisms of secondary brain injury after SAH are multifactorial. Although the incidence of vasospasm was successfully decreased in clinical trials, the translation into improved functional outcome failed [4]. In the last years, the focus of experimental and clinical research shifted towards pathophysiologic mechanisms in the first 72 h after the bleeding, commonly referred to as “early brain injury” (EBI). EBI was first described in 2004 by Kusaka et al. [5] and is more and more recognized as an important denominator related to delayed cerebral ischemia (DCI) and long-term morbidity and mortality after SAH [6••, 7].

In this review, we aimed (1) to summarize the current understanding of underlying pathophysiologic mechanisms of EBI, (2) to comment on various definitions of EBI commonly used in literature, and (3) to discuss current and potential future treatment implications. We decided to focus on available studies in humans after SAH to make this review suitable for clinicians in the care of SAH patients.

### Mechanisms of EBI

**Fig. 1:**
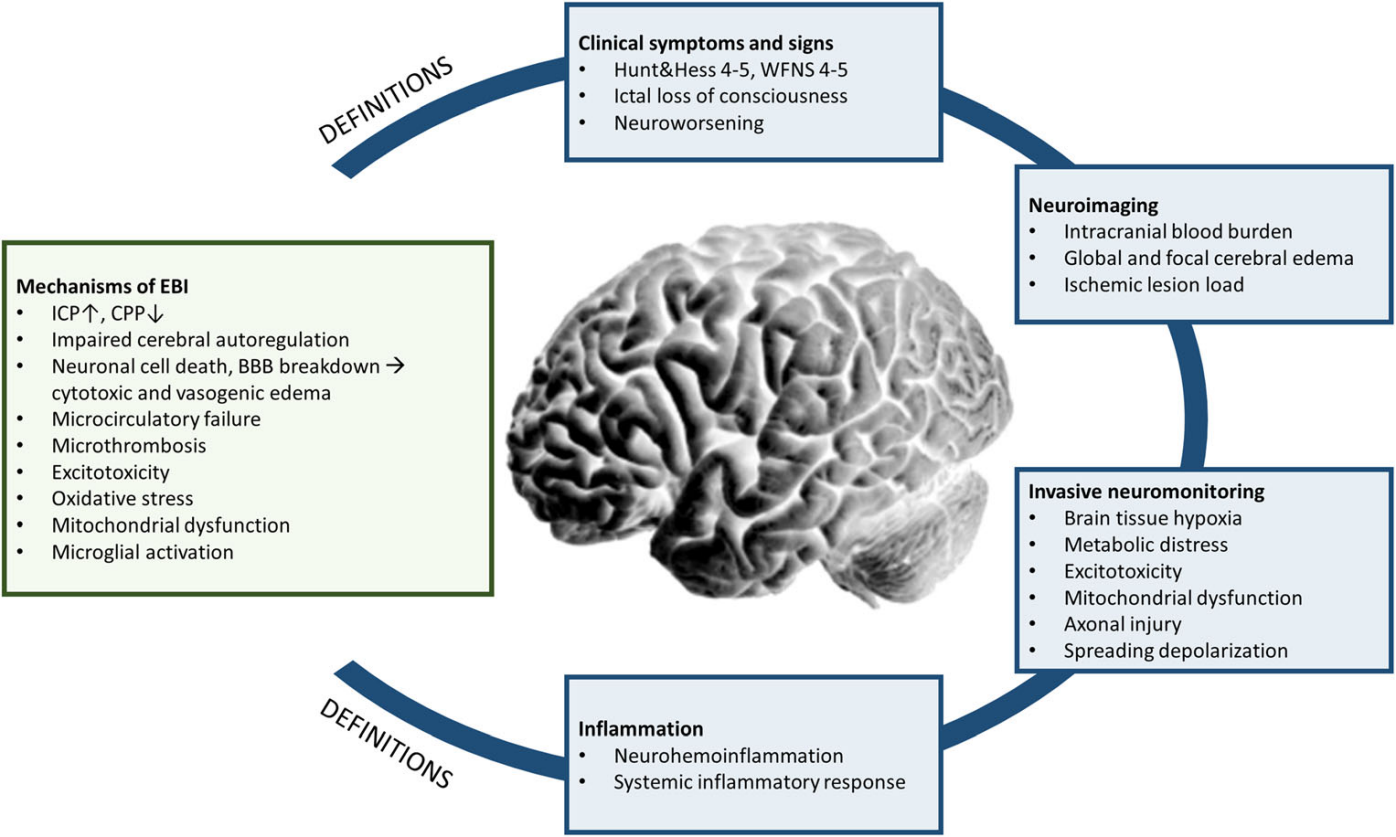
The complex pathophysiologic mechanisms contributing to early brain injury (EBI) after subarachnoid hemorrhage (SAH). Definitions of EBI used in the clinical setting are heterogenous and include clinical symptoms, neuroimaging parameters, and advanced neuromonitoring techniques. ICP = intracranial pressure, CPP = cerebral perfusion pressure, BBB = blood-brain barrier

There is increasing evidence that pathophysiologic mechanisms of brain injury start immediately after the bleeding. A sudden increase in intracranial pressure (ICP) caused by the extravasation of blood in the subarachnoid space provokes a decrease in cerebral perfusion (cerebral perfusion pressure, CPP), impairment of autoregulation [8•], and in severe cases, transient or persistent ischemia [9]. Neuronal cell death and endothelial damage result in cytotoxic edema and blood-brain barrier (BBB) breakdown [10–12], which aggravates the development of vasogenic edema [13, 14]. Cell death is furthermore believed to be a consequence of microcirculatory failure, microthrombosis, altered ionic homeostasis, excitotoxicity, oxidative stress, and neuronal swelling [7, 15]. Besides ischemia, “nonischemic” mechanisms such as energy dysfunction secondary to cortical spreading depolarizations (SDs) [16] or mitochondrial dysfunction [17] are also considered to be an important mitigator of EBI [18•]. Finally, the blood in the subarachnoid space and intracerebral hemorrhage itself may aggravate brain injury by microglial activation and initiation of a proinflammatory response [19] (Fig. 1).

## Definitions

So far, no consensus exists on a uniform definition of EBI which has led to an uncritical use of the term “EBI” in clinical practice and research. In the following section, we aimed to describe different approaches used for the definition of EBI based on clinical parameters, neuroimaging, and advanced neuromonitoring techniques (Fig. 1).

### EBI Based on Clinical Signs and Symptoms

Clinical evaluation of disease severity soon after the bleeding is a mainstay in the definition of EBI. Commonly used grading scales include the Hunt&Hess grade [20] or WFNS scale [21]. Poor-grade patients (commonly defined as H&H or WFNS grade 4–5) with prolonged loss of consciousness [22••] more likely suffer from EBI. It remains questionable whether patients with early obstructive hydrocephalus who mimic a severe disease and rapidly improve after the insertion of an external ventricular drain also qualify for this definition [23]. Together with a poor clinical grade on admission, ictal loss of consciousness (LOC) may best reflect EBI as a clinical equivalent of global cerebral ischemia or progressive intracranial hypertension [22••, 24]. In this context, it is important to mention that early-onset seizures with prolonged time to regain consciousness may again mimic EBI leading to a misclassification of the clinical grade soon after the bleeding. This is supported by the finding that SAH patients with early-onset seizures more often achieve good outcomes as compared with poor-grade patients without early seizures [25]. Still, early-onset seizures may also aggravate EBI, especially if seizures are accompanied by hemodynamic instability and increased energy demand.

Another clinical manifestation suggestive of EBI is early neuroworsening. This may be associated with a high blood burden in the subarachnoid, intraventricular or intraparenchymal space [26], and other factors such as rebleeding [27] early obstructive hydrocephalus, and early-onset seizures.

### EBI Based on Neuroimaging

One important neuroimaging biomarker of EBI is the amount of the intracranial blood volume early after the bleeding. Semiquantitative grading scales evaluating the blood burden in the subarachnoid space and the presence of intraventricular or intraparenchymal bleeding are commonly integrated in the prediction of DCI and outcome after SAH [28–30]. The association of a higher intracranial blood volume with poor clinical grades and poor outcome [26]suggests its significant contribution to the pathophysiologic concept of EBI. A more sophisticated approach to quantify the amount of blood in the subarachnoid space using a semiautomated process was not better in the prediction of hospital complications and poor outcome [31].

Another commonly used neuroimaging biomarker to EBI is the presence of brain edema early after SAH. While admission global cerebral edema (GCE) correlates with early disease severity and links EBI with secondary brain ischemia and poor outcome, this construct lacks sensitivity in the identification of unilateral or focal brain edema [32, 33]. More recently, a simple semiquantitative score grading both focal and global brain edema (SEBES, Subarachnoid Hemorrhage Early Brain Edema Score) was introduced [6••]. The SEBES is a CT-based evaluation of the absence of visible sulci caused by effacement of sulci or disruption of the gray-white matter junction at 2 predefined brain tissue levels (basal ganglia and centrum semiovale) in each hemisphere. The score ranges from 0 (no edema) to 4 (GCE) and may therefore better describe the transition from focal edema to global brain swelling. Admission high-grade SEBES (3–4 points) was furthermore associated not only with clinical disease severity but also with DCI and poor functional outcome [6••]. Moreover, GCE was quantified by selective sulcal volumes (SSV) using a semiautomatic approach. Smaller SSV suggestive of GCE were also associated with worse outcomes and may be a candidate marker of EBI [34].

Another way to quantify parenchymal pathologies and therefore EBI with advanced neuroimaging techniques comprises the early use of brain MRI by identifying the ischemic lesion load as well as vasogenic and cytotoxic edema [14, 35, 36].

In summary, neuroimaging techniques are useful to quantify EBI in a standardized way and may help to discriminate patients with a higher injury load early after the bleeding. Modern MRI techniques and the use of serial imaging may further elucidate the amount of axonal damage and the result of microvascular ischemic injury after SAH.

### EBI Based on Invasive Neuromonitoring

Invasive multimodal neuromonitoring techniques provide a unique opportunity to monitor pathophysiologic mechanisms of EBI at the cellular level in unconscious patients with SAH [37••]. It is important to mention that the selection of poor-grade patients for invasive monitoring limits the generalizability of these results to intermediate- or good-grade patients. The interpretation of study results using invasive neuromonitoring techniques is further limited by on the lack of high spatial resolution because neither unilateral hemispheric damage nor the global pathology may be identified. Integration of the probe location is therefore of paramount importance in the interpretation of brain oxygenation, CBF (cerebral blood flow), and metabolic changes [38]. Analysis of trend statistics is also useful to overcome this limitation and to early identify tissue at risk before a permanent damage occurs [39]. Another noteworthy limitation of data interpretation in the early phase of monitoring is the risk of insertion injury during probe placement. Therefore, the initial hours of monitoring should be interpreted with caution.

So far, only single-center observational trials investigated the early phase after SAH, mostly when the aneurysm has already been secured [18•, 37••, 40, 41]. These data suggest that brain oxygenation is diminished in the early phase after the bleeding.

Reasons for this can be multifactorial, including a compromised oxygen delivery secondary to an increase in ICP, stunned myocardium with decrease in cardiac output, and an increased brain oxygen consumption. The metabolic correlate can be quantified by an increase in the lactate-to-pyruvate ratio (CMD-LPR), a decrease in brain tissue glucose, and a profound excitatory response [37••]. Cell damage may be assessed by increased CMD-glycerol levels [41]. In a study including 18 poor-grade SAH patients, brain metabolic changes in the first 72 h revealed elevated levels of CMD-LPR, CMD-glutamate, and CMD-glycerol in the presence of a normal or even hyperemic state, evaluated by brain computed tomography (CT) perfusion scans [18•]. This finding is important and implicates that impaired cerebral metabolism explaining EBI is not only a consequence of brain tissue hypoxia but also reflects nonischemic metabolic distress including mitochondrial dysfunction [42].

Cerebral microdialysis furthermore allows the analysis of brain extracellular biomarkers, e.g., of axonal injury or neuroinflammation. Recently, higher levels of brain extracellular CMD-total-TAU protein indicating severe axonal damage were linked to poor neurologic outcome at 1 year [43]. These results highlight the potential of this method in further understanding mechanisms of EBI.

### EBI Based on Neurohemoinflammation and Systemic Inflammation

There is increasing evidence that neurohemoinflammation occurs early after SAH and is one potential mechanism of EBI [37••]. In a multimodal neuromonitoring study including 26 poor-grade SAH patients, the proinflammatory cytokine CMD-IL6 was highest in the initial phase after SAH and higher in patients with aneurysm rebleeding, GCE, and episodes of CPP < 70 mmHg [37••]. The proposed pathophysiologic mechanism includes early brain edema secondary to the disruption of the BBB and neuronal apoptosis by proinflammatory cytokines [10, 44]. Concordantly, CMD-MMP-9 levels were upregulated in the first 12 h after monitoring start, especially in patients with LOC, poor clinical grade, and initial brain tissue hypoxia [37••]. MMP-9 is known to be involved in endothelial basal membrane damage, neuroinflammation, and apoptosis and may therefore play an important role in the pathogenesis of EBI [45]. Neither CMD-IL6 nor CMD-MMP-9 was associated with systemic inflammatory markers underlining the idea of compartmentalization of the central nervous system. Still, there is evidence that also increased peripheral levels of cytokines are associated with EBI [46••]. In specific, IL-6, IL-10, and MIP1ß have recently been identified as an indicator of EBI using correlation network analysis in humans [46••]. Moreover, early platelet activation, systemic inflammation, and SIRS (systemic inflammatory response syndrome) are common in the early phase after SAH and associated with disease severity and poor functional outcome [47, 48•].

### EBI Based on Cortical Spreading Depolarizations

Recent evidence suggests that electrical brain failure may contribute to brain tissue injury after SAH [49••, 50••]. Cortical spreading depolarizations (SDs) are self-propagating waves of neuronal and glial electrical depolarization [51], which can be recorded using subdural strip electrodes in humans [52]. Although SDs may be associated with vasodilation in healthy subjects, their occurrence after SAH is commonly associated with inverse neurovascular coupling leading to hypoperfusion, brain tissue hypoxia, and metabolic derangement. SDs have been identified as a determinant of EBI in patients after SAH although the proof of causality needs further confirmation. In a recent study including 23 poor-grade SAH patients, the presence of ischemic and/or hemorrhagic lesions in the frontal cortex was associated with a higher incidence of SDs [49••]. The association between SDs and early focal brain injury is further supported by a larger study conducted by Eriksen et al. [50••]. While 33/37 (89%) patients with early focal brain injury exhibited SDs in the first 4 days, only 7/17 (41%) patients without early focal brain injury had SDs. Moreover, SDs correlated with the volume of early focal brain injury during the acute phase after SAH in this study.

### EBI Based on EEG Findings

Immediate or early-onset seizures commonly described as convulsive seizures occur at ictus and within the first 12 to 24 h after SAH [53]. Convulsive seizures in the very early phase were reported in 4.8% of SAH patients in a systematic review including 14 studies [53]. Although not entirely understood, they may be triggered by transient biochemical changes following the bleeding. Associated factors with ictal and early seizures include raised ICP, direct toxicity of the blood, neuroinflammation, vasospasm, and SDs. From a clinical point of view it can be difficult to differentiate between true seizures and nonepileptic movements related to raised ICP or herniation in the initial phase after SAH. As already mentioned, clinical reevaluation after prehospital seizures is important since seizures may lead to misclassification of clinical grades. The value of continuous EEG (cEEG) monitoring in the early phase after SAH needs further confirmation based on prospective multicenter studies. The primary goal of cEEG monitoring in SAH patients lies in the detection of subclinical seizures and nonconvulsive status epilepticus (NCSE) [54]. Around 3 to 19% of SAH patients develop NC seizures and 11% have NCSE [55–57].

## Current Implications for Clinical Practice

It is important to separate primary brain damage from mechanisms leading to secondary brain injury, which are potentially amenable to specific treatment strategies. Currently, there is no therapy available specifically targeting EBI. Management strategies to ameliorate EBI in the early phase after SAH primarily aim to provide sufficient energy supply to the brain and normalizing pathological parameters which are known to interfere with aggravation of EBI.

### ICP Control

Early intracranial hypertension is strongly interrelated with the pathogenesis of EBI and can result from multiple factors including GCE, acute hydrocephalus, intraparenchymal hematoma, and intraventricular hemorrhage. A stepwise approach to decrease ICP depending on the need of neurosurgical interventions and other common treatment strategies should be followed to decrease the risk of brain injury. Recent studies suggest that the amount of raised ICP above a certain threshold (ICP-burden) is more important than absolute ICP levels [58].

Approximately 50% of patients are admitted with obstructive hydrocephalus [59] and urgently need sufficient cerebrospinal fluid (CSF) drainage [60, 61]. Although the early development of hydrocephalus may not primarily be regarded as a marker of EBI, inadequate or delayed treatment can easily aggravate EBI and result in secondary brain damage. Other interventions to decrease ICP include optimal positioning of the patient, ventilation strategies targeting normocapnia or short-term hyperventilation, adequate sedation, and analgesia and surgical hematoma evacuation in case of a mass lesion [7, 62]. Osmotherapy is commonly used to decrease ICP although the effect on outcome is less clear. Only case series addressing the impact of hypertonic saline on ICP treatment could be identified with solely one study showing outcome improvement in poor-grade SAH patients [63]. In a multimodal neuromonitoring study, a potential benefit of normothermia due to lowering of ICP and ameliorating metabolic distress was suggested [64]. As a last tier treatment option of refractory ICP, hypothermia [65], barbiturate coma, or decompressive craniectomy [66, 67] may be considered.

### CPP Optimization

The optimal range of CPP levels in different phases after SAH is less clear. While a systolic blood pressure below 140 mmHg should be targeted before aneurysm obliteration, permissive hypertension is recommended when DCI is diagnosed [60, 61]. It is important to mention that a CPP targeted management with pressure calibration at the level of foramen of Monro is recommended when ICP is measured continuously. Recent data derived from observational studies using invasive multimodal monitoring techniques suggest that a higher CPP (> 70 mmHg) may ameliorate brain tissue hypoxia and metabolic distress in poor-grade SAH patients [37••, 68]. Still, a large interindividual variability may exist depending on the status of cerebral autoregulation (CA). CA is commonly impaired in the early phase after SAH, although recent data argue against the use of a predefined cutoff level for diagnosis [8•]. Systemic application of erythropoietin within 72 h of bleeding shortened the time with impaired autoregulation and reduced the risk of developing DCI after SAH in a prospective randomized trial (EPO trial) [69]. Because evidence of individualized CPP targets based on the CA status in acutely brain-injured patients is lacking, a phase II trial is currently investigating the safety and feasibility of such an approach in traumatic brain injury (TBI) patients (COGITATE trial: NCT02982122).

Further studies are needed to support the idea of individualized CPP goals as part of personalized medicine integrating the information of multimodal neuromonitoring techniques in the management of poor-grade SAH patients at high risk of EBI.

### Management of GCE

There is limited literature how to best treat patients with admission GCE or high-grade SEBES. Admission GCE was associated with hypermetabolism and metabolic distress arguing for interventions targeting at sufficient energy supply and amelioration of metabolic demand [33, 70]. In this line, CPP levels above 90 mmHg were associated with improved brain metabolism in an observational trial in patients with GCE [33]. Further studies are needed to support such an invasive treatment approach. Another potential intervention to target GCE is hypothermia [71], which is endorsed by animal data and needs confirmation in clinical trials. For now, targeted temperature management (TTM) in the sense of normothermia is commonly applied in many centers taking care of SAH patients (see next paragraph) [72].

### Targeted Temperature Management

Up to 60% of critically ill brain-injured patients experience fever during the first 24 h after admission [73]. Based on the known deleterious effect of fever on outcome [73, 74], normothermia is currently recommended [75, 76]. So far, there is no high-grade evidence that normothermia improves outcome in SAH patients. A prospective, randomized, multicentre study currently investigates the impact of normothermia on functional outcome (INTREPID: NCT02996266) after acute brain injury including SAH. As mentioned previously, hypothermia was only tested in the setting of refractory ICP and GCE in small single-center trials in combination with barbiturate coma [65, 71].

### Management of Intracerebral Blood Burden

Since the intracranial blood burden is associated with poor outcome, trials aimed at aggressive neurosurgical clearance of blood with conflicting results which is therefore not recommended by guidelines [60, 61]. There is some evidence that a reduction of the postoperative clot volume may be associated with a reduction in secondary complications, including DCI; however, this approach has to be tested prospectively [77].

### Management of Cortical Spreading Depolarizations

Addressing the cessation of SDs remains challenging and raises questions about the optimal pharmacological agent. Case reports and a retrospective review [78] suggest that ketamine has a dose-dependent suppressive effect on SDs. A first prospective small pilot study in ten TBI and SAH patients confirmed an effective inhibition of SDs by ketamine over wide ranges of dosage [79]. The effect on outcome improvement needs further investigations. Other potential treatment candidates include hypothermia and nimodipine. For now, targeting normothermia and normotension and providing sufficient energy supply to the brain are recommended by experts since fever, hypotension, and hypoglycemia may trigger SDs in acutely brain-injured patients [80].

### Management of Seizures

Antiepileptic therapy should be initiated in patients with seizures; however, a prophylactic use is not recommended [60, 61].

### Treatment of Aneurysms

Optimal patient management with early aneurysm securing aims at the prevention of rebleeding [60, 61]. Most of rebleeding events occur in the very early phase after SAH and result in poor functional outcome or death [27]. Although early obliteration of aneurysms might be beneficial, conflicting data exist for best timing of aneurysm treatment [81].

### Management of Neuroinflammation

Several agents including ASS, NSAIDs, thromboxane synthase inhibitors, steroids, nitric oxide donors, and immunosuppressant therapies have been tested to treat neuroinflammation after SAH; however, none of these drugs can be recommended as routine treatment so far [82••]. In a multimodal neuromonitoring study including 24 poor-grade SAH patients, brain interstitial CMD-IL 6 levels decreased following the administration of diclofenac [83]. These findings suggest a positive effect of parenteral diclofenac on the extracellular proinflammatory response in these patients.

## Conclusion

In summary, pathophysiologic mechanisms in the first 72 h after SAH gained clinical and research interest in the past decade. Based on the association of a more severe injury pattern early after the bleeding with secondary complications and poor outcome, future research should focus on EBI by integrating brain-derived biomarkers in order to improve the critical care management after SAH. An agreement on the use of a common definition is strongly needed.
